# Examining the role of common and rare mitochondrial variants in schizophrenia

**DOI:** 10.1371/journal.pone.0191153

**Published:** 2018-01-25

**Authors:** Vanessa F Gonçalves, Stephanie N. Giamberardino, James J. Crowley, Marquis P. Vawter, Richa Saxena, Cynthia M. Bulik, Zeynep Yilmaz, Christina M. Hultman, Pamela Sklar, James L. Kennedy, Patrick F. Sullivan, Jo Knight

**Affiliations:** 1 Department of Psychiatry, University of Toronto, Toronto, ON, Canada; 2 Campbell Family Mental Health Research Institute, Centre for Addiction and Mental Health, Toronto, ON, Canada; 3 Department of Genetics, University of North Carolina, Chapel Hill, NC, United States of America; 4 Functional Genomics Laboratory, Department of Psychiatry and Human Behavior, University of California, Irvine, CA, United States of America; 5 Center for Genomic Medicine, Massachusetts General Hospital, Boston, MA, United States of America; 6 Department of Psychiatry, University of North Carolina, Chapel Hill, NC, United States of America; 7 Department of Nutrition, University of North Carolina, Chapel Hill, NC, United States of America; 8 Department of Medical Epidemiology and Biostatistics, Karolinska Institutet, Stockholm, Sweden; 9 Division of Psychiatric Genomics, Department of Psychiatry, Institute for Genomics and Multiscale Biology, Icahn School of Medicine at Mount Sinai, New York, NY, United States of America; 10 Institute of Medical Science, University of Toronto, Toronto, ON, Canada; 11 Biostatistics Division, Dalla Lana School of Public Health, University of Toronto, Toronto, ON, Canada; 12 Data Science Institute and Medical School, Lancaster University, Bailrigg, Lancaster, LA1 4YW, United Kingdom; Kunming Institute of Zoology, Chinese Academy of Sciences, CHINA

## Abstract

Oxidative phosphorylation within mitochondria is the main source of aerobic energy for neuronal functioning, and the key genes are located in mitochondrial DNA. Deficits in oxidative phosphorylation functioning have been reported for schizophrenia, but efforts in the identification of genetic markers within the mitochondrial DNA that predispose to schizophrenia have been limited. We genotyped a set of mitochondrial SNPs using Illumina HumanExome arrays and tested for association in the Swedish schizophrenia sample (N> 10,000). We developed a novel approach for mitochondrial DNA imputation in order to increase the number of common SNPs available for association analysis. The most significant findings were for the mitochondrial SNPs C15452A (GRCh38.p10; rs527236209; p = 0.007; gene MT-CYB; defining haplogroup JT); A11251G (rs869096886; p = 0.007; gene MT-ND4; defining haplogroup JT), and T4216C (rs1599988; p = 0.008, gene MT-ND1, defining haplogroup R2’JT). We also conducted rare variant burden analyses and obtained a p-value of 0.007. For multimarker haplotypes analysis, the most significant finding was for the J group (OR: 0.86, p = 0.02). We conducted the largest association study of mitochondrial DNA variants and schizophrenia but did not find an association that survived multiple testing correction. Analysis of a larger sample is required and will allow a better understanding of the role of mitochondria in schizophrenia.

## Introduction

Schizophrenia (SCZ) is a complex disorder characterized by psychosis and disturbed behavior. The heritability of SCZ is estimated at 80% [1] and although substantial progress has been made [1,2], the genetic risk factors are still far from being fully understood.

Oxidative phosphorylation within mitochondria is the main source of aerobic energy for neuronal functioning. Genes within the pathway are encoded by the nuclear genome (approximately 95 genes) as well as by mitochondrial DNA (13 genes). Impairments in oxidative phosphorylation might be involved in the pathophysiology of diseases with bioenergetic deficits. Deficits in brain energy metabolism have been noted in SCZ, and mitochondrial dysfunction in SCZ has been proposed (reviewed by Gonçalves et al. [3]).

Genetic evidence has suggested that nuclear-encoded mitochondrial genes may play a role in SCZ [1], although the mitochondrial related pathways were not significantly associated with the disease in a recent large study (N = 9,379 SCZ cases) [4]. There are a limited number of association studies between mitochondrial DNA and schizophrenia, and the results have been conflicting [3]. Some studies focused on the association of mtDNA haplogroups (i.e., clusters of mitochondrial sequences sharing a specific set of polymorphisms as consequence of having a common ancestor). Positive findings were reported between haplogroup HV and SCZ in an Israeli sample (N = 220 cases) [5]; and between haplogroup J-T and onset of SCZ in an Italian sample (N = 142 cases) [6]. Hudson et al [7] found nominally significant associations between SCZ and SNPs defining haplogroup U in a data set based on genotyped and imputed SNPs (N = 2,950 cases). Positive findings were also reported on non-Europeans, for example, a study in Han Chinese population showed an association between haplogroup B5a and SCZ risk in a sample size of 1,212 cases [8]. Wang et al. [9] analyzed haplogroups and control region sequence data in a Chinese Han population and they reported a nominal association between haplogroup N9a and SCZ (N = 298 cases).

However, more recent studies do not support these findings [10–13], although the approaches used to assign individuals into haplogroups varied across the studies. For example, Bertolin et al. [11] conducted a case–control study in very small Italian SCZ and bipolar sample (N = 89 cases), and did not observe a difference in haplogroup frequencies between cases and controls. Mosquera-Miguel [12] genotyped 16 tag SNPs in two Spanish sample, and reported no association between major European haplogroups and SCZ (N = 942). Torrel et al [13] also reported negative findings for haplogroups HV, JT and U in a Spanish population (N = 495 cases). For non-Europeans, Ueno et al. [10] analyzed sequences of 93 SCZ Japanese subjects and compared frequencies of haplogroups with healthy controls. They did not find a significant difference between the two groups. Xu et al [14] performed restriction fragment length polymorphism PCR (PCR-RFLP) using four mtDNA SNPs in the coding region as well as sequencing of the control region to analyze haplogroups and variants in a Han Chinese case-control sample (313 cases). They did not find any significant association between variants or haplogroups and SCZ risk.

Two studies, the largest to date, with approximately 3,000 cases each and partially overlapping samples [7,15], investigated SNPs and found nominal associations. A study in Han Chinese families reported the presence of private non-synonymous variants in probands with SCZ [16]. Here, we tested the hypothesis that mitochondrial DNA variants play a role in SCZ. To the best of our knowledge, this is the largest study of its type.

## Methods

### Genotyping quality control

A comprehensive set of 220 mitochondrial SNPs were genotyped in one batch using Illumina HumanExome arrays and tested for association in a previously described Swedish SCZ case-control sample (N = 10,771) [2]. Samples that passed autosomal quality control procedures were selected (N = 4778 cases and N = 5819 controls) [17]. Additional quality control steps comprised removing individuals showing missing genotype rate greater than 5% for mitochondrial markers (N = 3). A total of 729 heterozygous haplotypes possibly due to mitochondrial heteroplasmy were coded as missing (final N = 4775 cases and N = 5819 controls). Genotype data were available for 42 coding region SNPs with a minor allele frequency (MAF) greater than 1% and 123 coding region SNPs with MAF ≤ 1%. We excluded SNPs in the control region (16024 to 576 bp) because there are no genes in this region (Information regarding the genotypes for common SNPs are described in the S1 Table).

### Haplogroup assignment

We used HaploGrep [18] to assign haplotypes to haplogroups and to check for potential contamination in our dataset. Briefly, HaploGrep weights each polymorphism present in Phylotree17 (a phylogenetic tree of worldwide human mitochondrial DNA variation) based on its informativeness to define haplogroups. The set of SNPs in the input file are classified as informative or remaining (not informative). A score is given based on the weights of the “informative SNPs” but it is “penalized” by the number of remaining SNPs (for details see (14)). There are eight main Europeans haplogroups under the N clade: HV (H, V), JT (J, T), U (which includes K), I, W, and X [19]. To check for contamination or uncertainty in the haplogroup classification (the last due to limited number of SNPs available in the genotyping chip), we attempted to assign haplogroups with the remaining SNPs. In clean data, the reference group H2a2a1 is expected, but if a different haplogroup is assigned, it suggests contamination. We found a second haplogroup for 3.5% of the sample and we excluded these individuals (N = 380) from further analysis. Thus, the total number of samples available for analysis was 10,214 (4,591 cases and 5,623 controls). We also excluded samples with imputed haplogroups with overall rank less than 0.8.

### Mitochondrial DNA imputation approach

We developed a novel approach for mitochondrial DNA imputation that allowed us to gain data for another 30 common SNPs giving us 71 in total (considering post-imputation filters of “info” score > 0.3 and MAF > 1%). We downloaded 7,141 public European mitochondrial sequences from Human Mitochondrial DataBase [20] and used them as reference panel (SNP N = 188 after filtered by MAF > 1%). Imputation was performed using IMPUTE2 v.2 software [21], following the instructions for chromosome X and recoding all individuals in Swedish data set as males for the purpose of this analysis (List of haplotypes with genotyped/imputed SNPs are in S3 Table). We evaluated imputation performance, both by removing genotyped SNPs one at a time from input files and confirming accuracy of imputed genotypes and by comparing allele frequencies of the imputed SNPs with reported frequencies from other datasets (see Results section).

To determine the accuracy of our approach to assign haplogroups based on genotypes and to check whether imputed data would more accurately identify haplogroups than genotyped-only SNPs (from the Illumina HumaExome array), we performed the following analysis: i) We determined which haplogroups were present in our data using HaploGrep2; ii) We then extracted the full profile for each of these from Phylotree17; iii) From these profiles we selected only SNPs present on the Illumina HumanExome array to create pseudo-samples; and iv) We then imputed back any missing data for these pseudo-samples. Then, we compared the Phylotree assigned haplogroup with the haplogroups defined based on imputed (genotyped/imputed SNPs) and genotyped-only SNPs.

## Statistical analysis

For single marker tests, associations with SCZ for the **71** common SNPs (genotyped and imputed) were tested using SNPTEST v2.8 with the frequentist test and expected method. We conducted rare variants analyses on the genotyped markers with sequence Kernel association test (SKAT) [22], using Davies method to compute the p-value (an exact method that computes the p-value by inverting the characteristic function of the mixture chi square distributions) and using weights from a beta function on the MAF (SKAT default weights).

Following haplogroup assignment, we performed multidimensional scaling analysis (MDS) and plotted the first two dimensions in order to visualize traditional mtDNA haplogroups. Association testing was performed using macro-haplogroups, in which some phylogenetically-related haplogroups were combined. We tested the association between the macro-haplogroups HV, J, T, and U and SCZ using logistic regression. Power calculation was performed using Quanto 1.2.4 [23].

## Results

A total of 194 SNPs were present in the imputation output of which 41 had been genotyped (one SNP was filtered out due to excessive missing data after removal of 381 individuals with uncertain haplogroup assignment) and 153 imputed (141 in the coding region). After filtering for info score ≥ 0.3 and MAF ≥ 1%, we retained 71 SNPs (41 genotyped and 30 imputed) in the coding region for univariate association analysis. The 71 SNPs available for analysis equated to 35 independent tests (determined using SNP Spectral Decomposition Lite) [24]. We applied a Bonferroni-corrected significance threshold of 0.001. For MAF ≥ 1%, this sample had 80% power to detect a genotype relative risk of 1.6 (additive model, α = 0.001). Power for genome-wide significance (α = 5x10^-8^) was lower (80% power for a relative risk of 2.0).

To validate our mtDNA imputation approach, we removed the genotyped SNPs one at a time from input files and we were able to impute 34 of them (total number was 41). Our approach was efficient with 96% accuracy (S1 Fig). Furthermore, comparative analysis showed similar allele frequencies between imputed and genotyped SNPs from our study with the Swedish data described by Saxena et al [25] (N = 1,010) (S2 Fig).

In order to check the accuracy of genotype-only (array) and genotype/imputed SNPs to assign to haplogroups, we compared the results using both approaches with the expected haplogroup based on Phylotree17. We observed 90% concordance between Phylotree expected haplogroup and haplogroup assigned by genotyped-only SNPs (array) versus 86% concordance with results from genotyped/imputed SNPs.

For single SNP analysis, the most significant findings were for SNPs C15452A (rs527236209; p = 0.007, gene MT-CYB), A11251G (rs869096886; p = 0.007; gene MT-ND4), and T4216C (rs1599988; p = 0.008; gene MT-ND1) (Table 1). All three SNPs are used the define haplogroup JT. We also conducted rare variants analyses on the genotyped markers (N = 123 SNPs, MAF ≤ 1%) using SKAT [22] and obtained a p-value of 0.007.

**Table 1:** Top association for single SNPs association analysis results.

SNP rs_number (GRCH38.p10)	HVGS code	BP	Risk Allele	Freq_A1 (cases)*	Freq_A1 (controls)*	OR (CI)	P-value
**rs527236209**	NC_012920.1:m.15452C>A	15452	A	7.2	9.9	0.86 (0.80 0.93)	0.007
**rs869096886**	no available	11251	G	7.2	9.9	0.86 (0.80 0.93)	0.007
**rs1599988**	NC_012920.1:m.4216T>C	4216	C	7.4	10.1	0.86 (0.80 0.93)	0.008

* Frequency in %.

**Fig 1:**
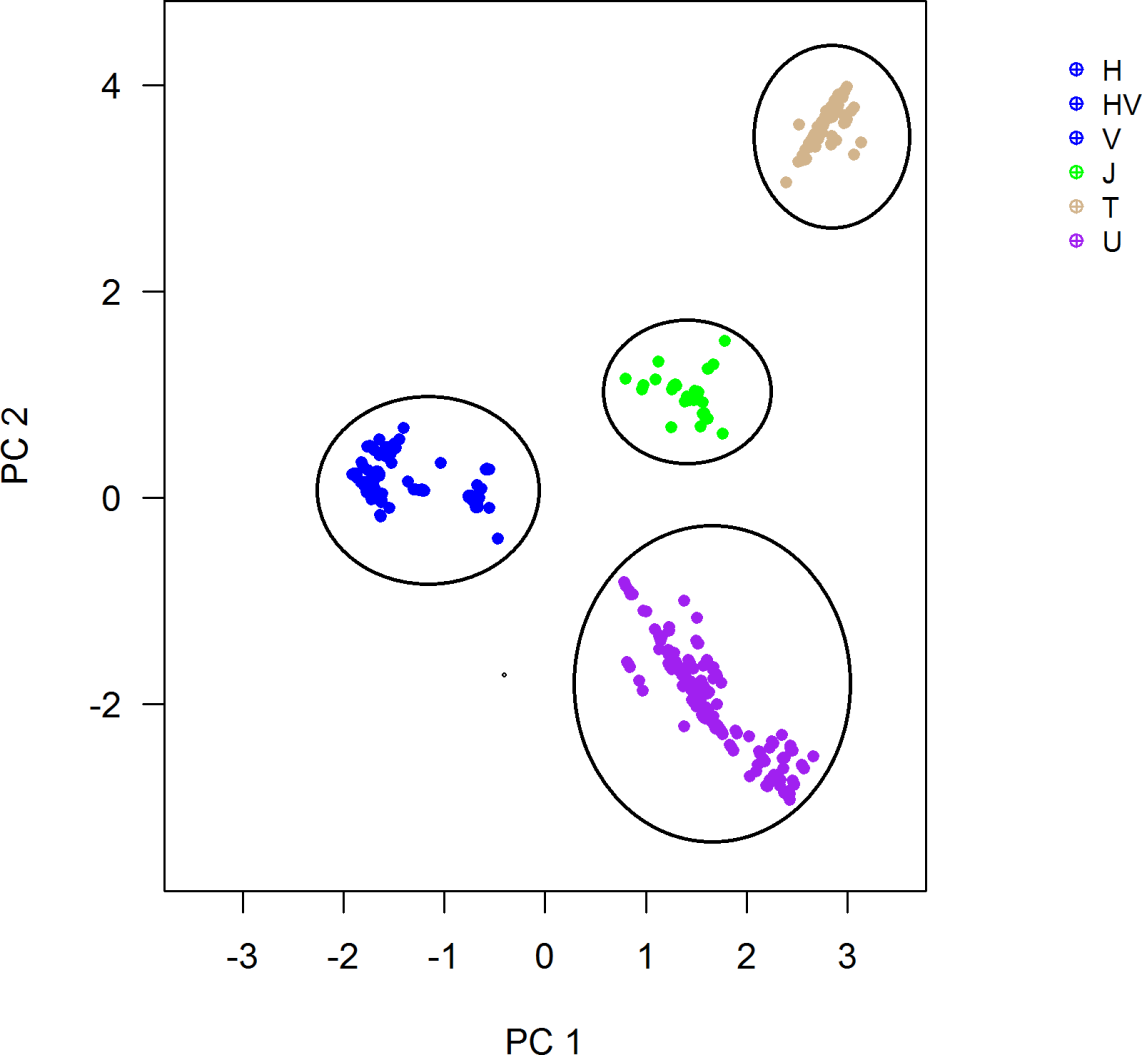
mtDNA genetic grouping. Colors correspond to the traditional mtDNA haplogroups according to HaploGrep2. The four clear groups defined by first and second dimensions are highlighted.

For analyses of haplogroup, we were able to assign most samples reliably into four European macro-haplogroups, HV, J, T, and U (Fig 1). Individuals assigned to the remaining European mtDNA haplogroups as well as non-European haplogroups were not included in our analysis. The most significant finding was for the J group (p = 0.02; 8.3% in cases and 9.7% in controls; OR: 0.86) (Table 2). The haplogroups identified in this study were listed in S2 Table.

**Table 2:** Top hits from mtDNA macro-haplogroup association analysis.

Haplogroups	% Controls	%Cases	p-value	OR (CI)
**J**	**9.7**	**8.3**	**0.02**	**0.86 (0.74–0.98)**
**H-HV-V**	**49.5**	**50.3**	**0.48**	**1.03 (0.95–1.11)**
**T**	**8.7**	**7.9**	**0.16**	**0.90 (0.78–1.04)**
**U(K)**	**25.1**	**25.9**	**0.37**	**1.04 (.095–1.14)**

## Discussion

In this study, we conducted the largest study to date investigating the role of mtDNA SNPs in the susceptibility to SCZ. None of the associations survived correction for multiple testing. However, we validated an imputation method for mitochondrial DNA that should improve our ability to identify associations in studies with more SNPs and/or samples.

We started with the set of 41 common genotyped SNPs and were initially only able to identify two (H and T) of the eight expected haplogroups (S3 Fig). The other groups were seen as one overlapped group, after the multidimensional scaling analysis. It was suggestive that important SNPs were missing for an accurate group analysis, and thus, we decided to impute mtDNA. We used standard approaches for imputation but adjusting for the unique features of mtDNA (such as the use of chromosome X protocol and removal of heterozygous genotypes). Using imputed mtDNA data, we were able to identify more clusters representing the haplogroups J, T, HV, and U. In future, haplogroup classification using microarrays could be improved by designing arrays that contain SNPs that are informative in relation to phylogeny. Noteworthy, in our current approach, we did not exclude hotspot variants from the imputed data before performing statistical analysis or haplogroup assignments. However, in our imputed data, we found only two hotspot variants (MT:5147 and MT:8251), as defined by Soares et al [26]. These variants have medium (4.6 and 4.5) HaploGrep2 weights respectively according to Weißensteiner et al [27] (the weight indicates the importance of the variant in the haplogroup assignment; lower values mean less influence on the classification by the tool—scale from 1-to-10). Thus, they do not have a strong influence on the haplogroup assignment.

The strongest findings for the single SNP analysis were for the SNPs C15452A (rs527236209); p = 0.007 (gene MT-CYB); A11251G (rs869096886); p = 0.007 (gene MT-ND4), and T4216C (rs1599988); p = 0.008 (gene MT-ND1). The three SNPs are used to define haplogroup J. We also found that the haplogroup J was the most strongly associated with SCZ.

This SNP C15452A (rs193302994) is a non-synonymous variant located in the Cytochrome b gene, which is the largest subunit of the oxidative phosphorylation complex III. The SNP A11251G (rs869096886) is a synonymous variant (syn L:L) located in the MT-ND4 gene (Mitochondrially Encoded NADH:Ubiquinone Oxidoreductase Core Subunit 4). Both SNPs define the haplogroup JT and its subclades (i.e., J and T haplogroups and their sub-haplogroups). The haplogroup JT was associated with age at onset in very small SCZ sample [6] but it was negative in two studies testing the association of mitochondrial haplogroups with SCZ [12,13]. The SNP T4216C (rs1599988) is a non-synonymous variant located in the MT-ND1 (mitochondrially encoded NADH:ubiquinone oxidoreductase core subunit 1). This SNP is key to define the pre-JT haplogroup and its subclades.

In conclusion, we did not find a significant association between mtDNA SNPs or haplogroups and SCZ. Variants with large effects are not expected to be found in the mitochondrial DNA due to fact that those variants (deleterious) are eliminated by the ovarian selection system [28]. Therefore, even a sample of >10,000 may not be adequately large to detect mitochondrial genetic variants that are associated with SCZ. Furthermore, a deeper analysis of rare mtDNA variants, particularly in larger samples, will shed light on the role of mitochondria in SCZ.

We were able to develop an imputation approach for mitochondrial DNA that will allow us to comprehensively analyze mitochondrial SNPs from groups such as the Psychiatric Genomics Consortium. Our study has some limitations: i) inability to assign people into all the haplogroups; however, assignment should be improved by increasing the current reference panel size for mitochondrial DNA imputation; ii) Our imputation approach does not outperform the haplogroup assignment by the genotyped-only SNPs (Illumina HumanExome array). Analysis of a larger sample is required and will allow a better understanding of the role of mitochondria in SCZ.

## Supporting information

S1 Fig Imputation accuracy. The axis Y shows concordance percentages between genotyped and imputed data sets for each of SNP present in the Illumina HumanExome arrays (coding region).
(TIF)

S2 Fig Comparison of imputed SNPs frequencies between our study and Saxena et al[25]. (TIF)

S3 Fig mtDNA genetic grouping using genotyped data only. Colors correspond to the traditional mtDNA haplogroups according to HaploGrep2. The three clear groups defined by first and second dimensions are highlighted.
(TIFF)

S1 Table Information regarding the genotypes for common SNPs present in the Illumina HumanExome arrays. (XLS)

S2 Table Haplogroups identified in this study using imputed mtDNA data. (XLSX)

S3 Table List of haplotypes (genotyped/imputed SNPs) found in this study. (XLS)
