# Supplementary figures and images for: Examining the role of common and rare mitochondrial variants in schizophrenia
Source: PLoS One. 2018 Jan 25;13(1):e0191153. doi: 10.1371/journal.pone.0191153 (PMC5784966; doi:10.1371/journal.pone.0191153)

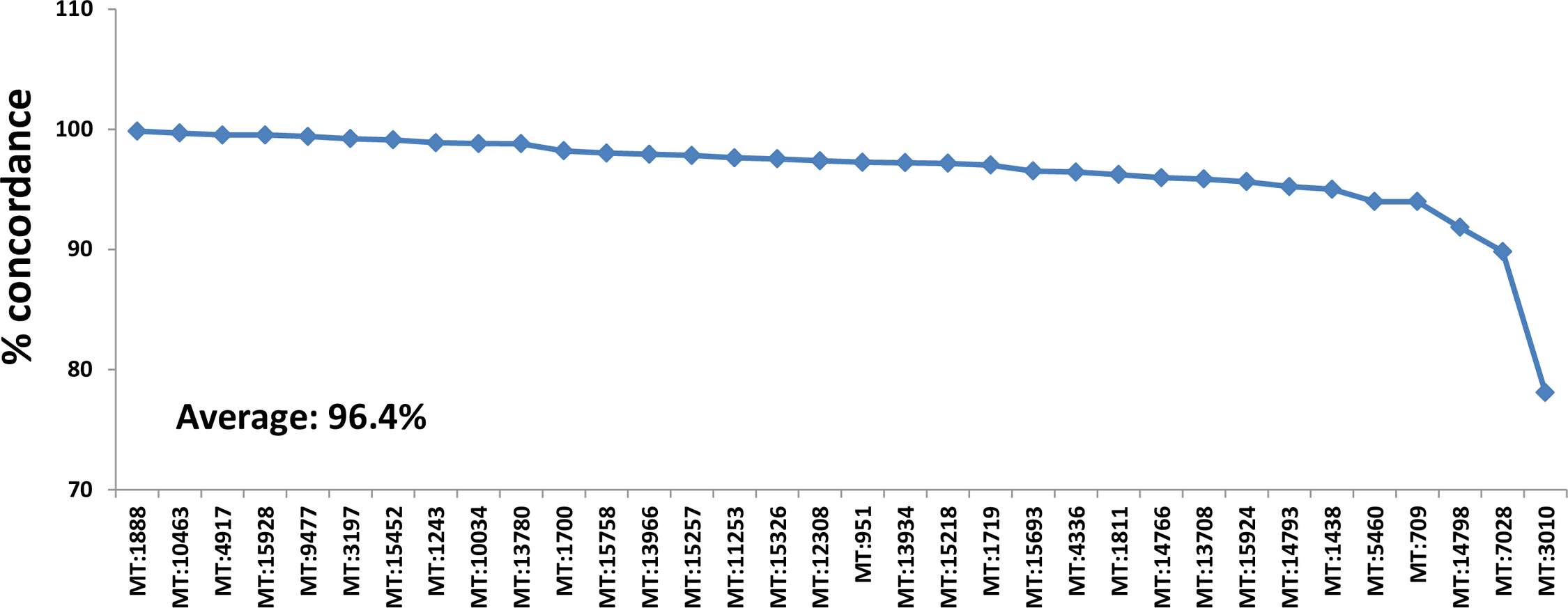

Supplement: S1 Fig — The axis Y shows concordance percentages between genotyped and imputed data sets for each of SNP present in the Illumina HumanExome arrays (coding region). (TIF) [file pone.0191153.s001.tif]

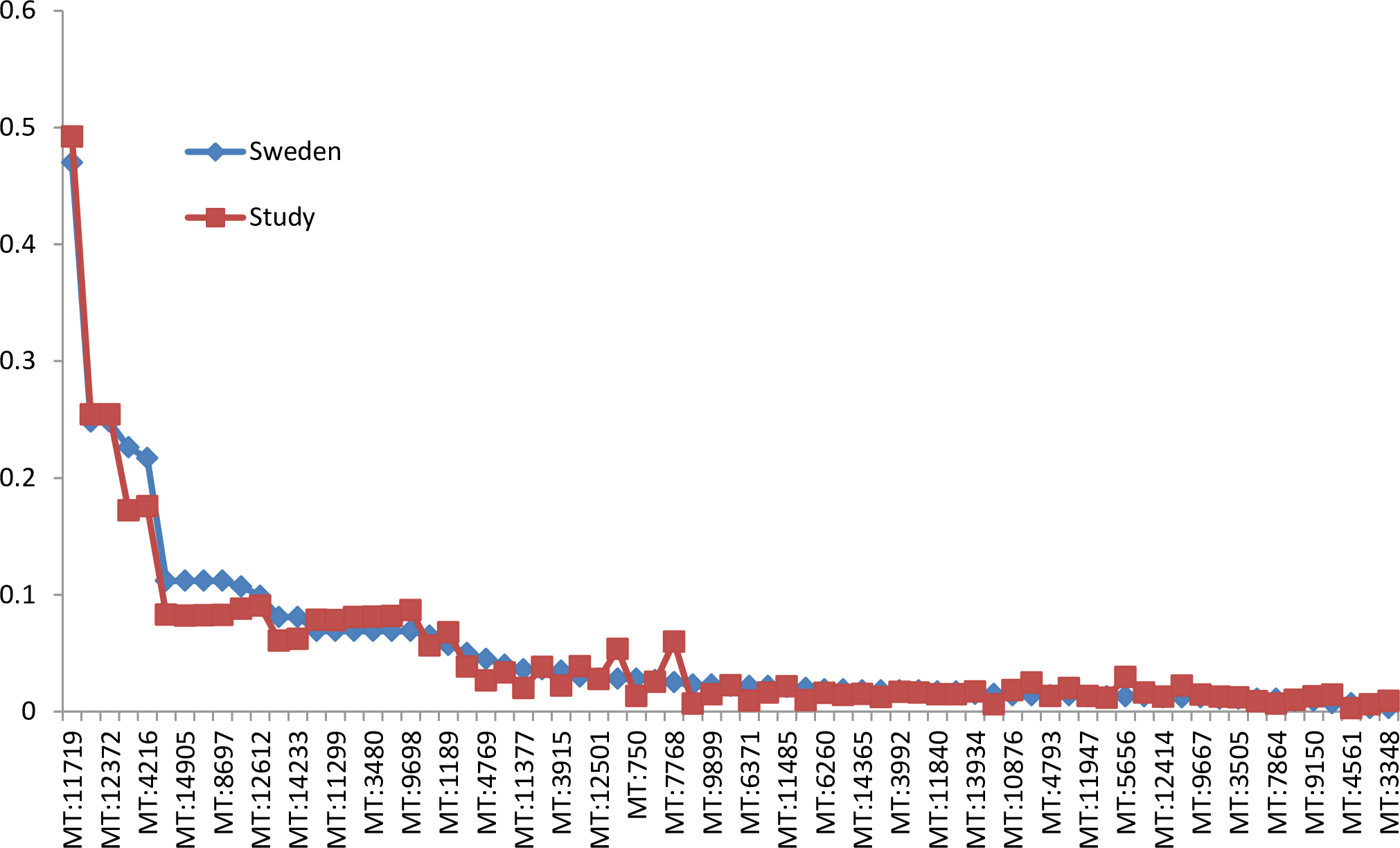

Supplement: S2 Fig — (TIF) [file pone.0191153.s002.tif]

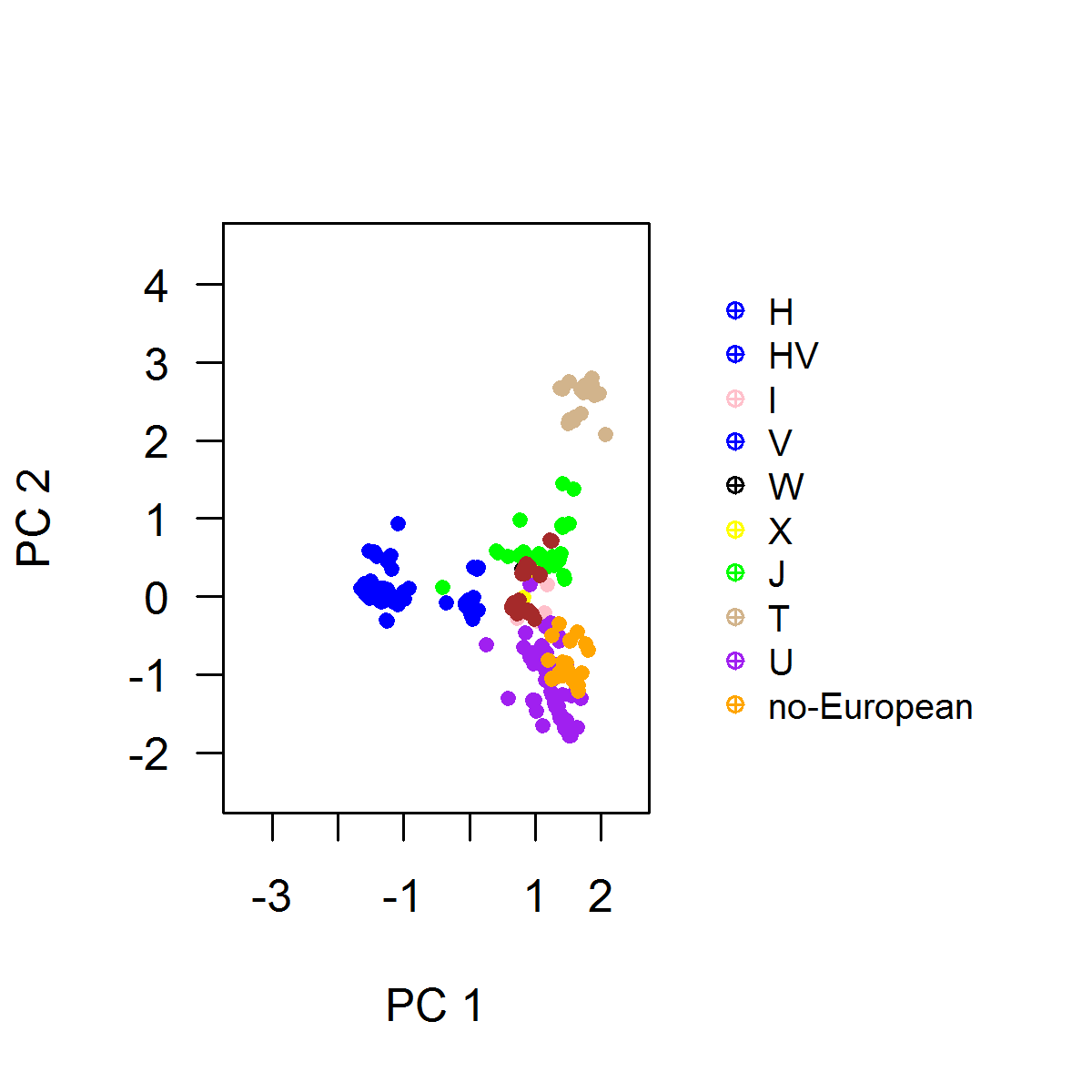

Supplement: S3 Fig — Colors correspond to the traditional mtDNA haplogroups according to HaploGrep2. The three clear groups defined by first and second dimensions are highlighted. (TIFF) [file pone.0191153.s003.tiff]
